# Lung cancer mortality attributable to residential radon in Germany

**DOI:** 10.1007/s00411-024-01095-y

**Published:** 2024-11-13

**Authors:** Felix Heinzl, Maria Schnelzer, Peter Scholz-Kreisel

**Affiliations:** Effects and Risks of Ionising and Non-Ionising Radiation, Federal Office for Radiation Protection, Ingolstaedter Landstr. 1, Oberschleissheim, 85764 Germany

**Keywords:** Population-attributable fraction (PAF), Residential radon, Lung cancer, Burden of disease, Health impact assessment

## Abstract

The radioactive gas radon is one of the most important risk factors for lung cancer after smoking. This article aims to estimate the annual number of lung cancer deaths attributable to residential radon exposure in Germany and its federal states using updated data and an advanced calculation method. Data on lung cancer mortality (2018–2022), smoking behavior (2017), and on the estimated distribution of radon concentration based on a radon residential study (2019–2021) in Germany are used. The risk model employed is derived from the pooled European residential radon study, indicating that excess relative risk for lung cancer increases by 16% per 100 becquerels per cubic meter (Bq/m$^{3}$) of corrected long-term radon concentration. It is estimated that a total of around 2800 lung cancer deaths per year (95% confidence interval (CI) 900–5100) are attributable to residential radon in Germany. This represents a population attributable fraction of 6.3% (95% CI 2.1–11.4%). Notably, radon-attributable lung cancer deaths occur not only among current (41%) but also significantly among former smokers (41%) and those who have never smoked (19%). The results confirm that radon in homes is an important risk factor for lung cancer, highlighting the need for protective measures against radon for all population groups in Germany.

## Introduction

The radioactive gas radon is a human carcinogen (IARC 1988) and is one of the most important risk factors for lung cancer after smoking (WHO 2023). An illustrative way of describing the lung cancer risk of residential radon is to give the so-called attributable lung cancer deaths caused by radon in homes. This indicator is valuable as it aids policymakers and the public in understanding the health impacts of radon exposure.

In 2006, it was estimated for the first time for reunified Germany that 1900 lung cancer deaths per year were attributable to radon exposure in homes (Menzler et al. 2008). Since then, some of the parameters used in calculating these attributable deaths have changed. For instance, between 2018 and 2022, an annual average of 44,900 people (17,200 women and 27,700 men) died from lung cancer in Germany. In contrast, the numbers used by Menzler et al. (2008), based on mortality data from 1996 to 2000, indicated about 37,700 annual lung cancer deaths (9200 women and 28,600 men). More recent statistics are also available regarding smoking behavior and the risk of lung cancer due to smoking; these have to be considered as radon and smoking interact and mutually increase lung cancer risk. New findings are also available on the radon distribution in Germany.

Thus, the objective of this study is to update the population-attributable fraction (PAF) and the number of lung cancer deaths attributable to residential radon in the German population. These indicators are calculated separately for six subpopulations—defined by sex and smoking. Contrary to the PAF approximation formula commonly used in the literature, which considers only the calculated mean radon exposure in a population, we adopt and refine the approach of Menzler et al. (2008). This method takes into account the entire distribution of radon exposure, yielding more precise results. Additionally, this detailed approach enables us to estimate the number of lung cancer deaths that could potentially be prevented through radon mitigation programs.

## Data and methods

The methodological approach primarily follows that of Menzler et al. (2008), incorporating updated data and some minor conceptual changes. All calculations and visualizations are performed using the R software (R Core Team 2022) and the programming environment RStudio (Posit team 2022).

### Mortality data

Like Menzler et al. (2008) and as is commonly done in the literature, we use mortality data instead of incidence data. This allows for the inclusion of un-diagnosed cases of lung cancer (death-certificate-only cases) and ensures consistency and comparability with previous analyses. The number of deaths from lung cancer (ICD-10: C33 malignant neoplasm of trachea and C34 malignant neoplasm of bronchus and lung), mortality rates for lung cancer and all causes of death were obtained from the causes of death statistics combined with population data, provided by the Federal Health Monitoring of Germany (GBE 2023). These data are available for Germany and its 16 federal states, and are divided by sex, age class (0, 1–14, 15–19,..., 85–89, 90+ years), and calendar year. The averages of the last five years (2018–2022) are used for the calculations in this publication. During this period, on average, 44,900 people (17,200 women and 27,700 men) died from lung cancer annually in Germany.

### Radon exposure data

**Fig. 1:**
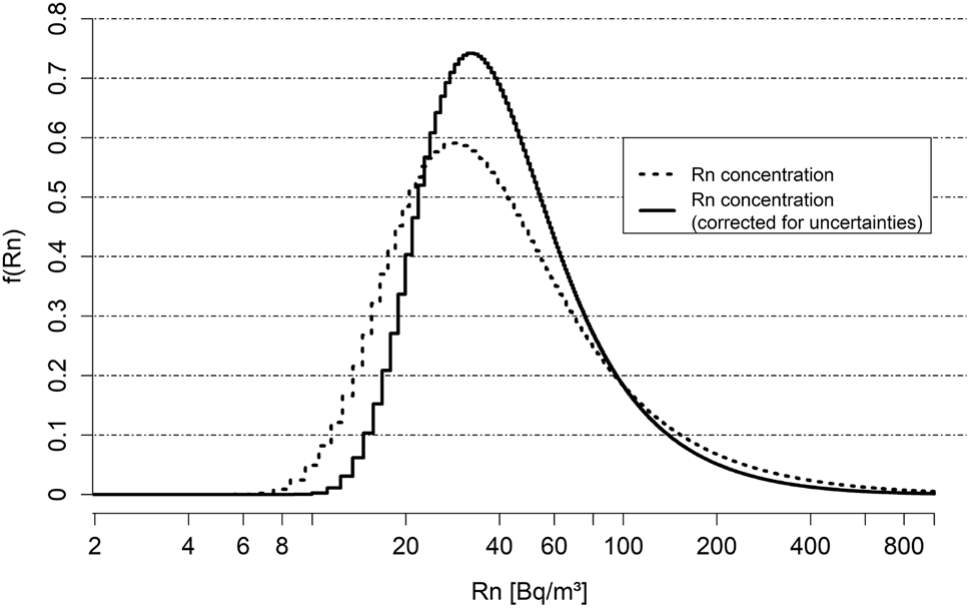
Density function of the corrected (solid line) and uncorrected radon concentration (dashed line) on a logarithmic scale for Germany

As in Darby et al. (2005), radon exposure is defined as the long-term time-weighted radon concentration over a period of 30 years. For the distribution of radon in Germany (shown in Fig. 1, dashed line) and its 16 federal states we use the results of Petermann et al. (2024). They predicted the radon distributions for each floor level of each residential building in Germany. These predictions were based on a modeling approach that utilizes environmental and building data, supplemented by 14,000 one-year measurements taken in 7500 households between 2019 and 2021 (Kemski et al. 2022).

The same methodology as in Darby et al. (2005) and Menzler et al. (2008) is employed to correct for measurement uncertainties. One of the primary sources of these uncertainties is the variability in one-year radon measurements, which can differ from year to year. Consequently, a 1-year measurement exhibits greater variability compared to a 30-year measurement. Therefore, the correction for measurement uncertainties leads to a lower arithmetic mean and to a shift of extreme values towards the mean (Table 1, Fig. 1).

**Table 1:** Characteristic values of the population-weighted radon distribution before (Petermann et al. 2024) and after correction for measurement uncertainties

Radon distribution	Means [Bq/m$^{3}$]		Quantiles [Bq/m$^{3}$]			Exceedance frequencies [%]			
	Arithmetric	Geometric	50%	90%	95%	100 Bq/m$^{3}$	300 Bq/m$^{3}$	600 Bq/m$^{3}$	1000 Bq/m$^{3}$
Before correction	63	41	36	115	180	12.5	2.2	0.7	0.3
After correction	55	43	39	98	141	9.8	1.0	0.2	0.1

### Relative risk for lung cancer by radon

To describe the relationship between lung cancer risk and residential radon exposure, the linear excess relative risk (ERR) model from the pooled European case–control study (Darby et al. 2005) is utilized. This study is the largest and most informative one worldwide to investigate the link between lung cancer and radon in homes. According to this model, the relative risk of lung cancer increases by 16% (95% confidence interval (CI) 5–31%) per 100 becquerels per cubic meter (Bq/m$^{3}$) of long-term residential radon concentration *x* after adjustment for radon measurement uncertainties. In this study, the exposure period was considered to be the period in the 5–34 years prior to the lung cancer diagnosis or the interview and thus a latency period of at least 5 years was assumed. Since people under the age of 35 years cannot have been exposed to radon for so long, their radon-induced lung cancer risk is lower:

1 $$\begin{aligned} & ERR_k(x) \approx \beta (a_k) \cdot x\nonumber \\ & \beta (a_k) = \left\{ \begin{array}{ll} 0, & a_k< 5 \\ \frac{a_k-5}{30} \cdot 0.16, & 5 \le a_k < 35 \\ 0.16, & a_k \ge 35 \\ \end{array} \right. . \end{aligned}$$

Here, $a_k$ denotes the midpoint of the age group *k*. Equation (1) was applied for all smoking groups as the ERR did not vary significantly among these groups in the European case–control study (Darby et al. 2006). This consistency suggests a multiplicative interaction effect between radon exposure and smoking on lung cancer risk (UNSCEAR 2020).

### Combined effect of smoking and radon

In contrast to Menzler et al. (2008), we differentiate among three smoking groups (current smokers, former smokers, and never smokers) to capture the interactions between radon and smoking on lung cancer risk more precisely. The categorization of smoking groups was based on the German microcensus (Destatis 2018): Individuals who currently smoke (whether regularly or occasionally) are classified as current smokers (S). Former smokers (F) are those who no longer smoke, and never smokers (N) are individuals who have never smoked, neither regularly nor occasionally. The distribution of lung cancer mortality rates and the number of lung cancer deaths among these smoking groups is conducted in accordance with common practices in the literature (Menzler et al. 2006; Bochicchio et al. 2013; Ajrouche et al. 2018; Kurkela et al. 2023). This split is applied to both male and female lung cancer rates as well as the number of deaths per age group. For this analysis, we use data on smoking behavior, separated by federal state, sex, and age group, from the German microcensus in 2017 (Destatis 2018) (Table 2).

**Table 2:** Smoking status percentages [%] for women and men by age group in Germany (Destatis 2018)

Age group [years]	Women			Men		
	Never smoker	Former smoker	Current smoker	Never smoker	Former smoker	Current smoker
0–14	100	0	0	100	0	0
15–19	90	1	9	86	1	13
20–24	75	5	20	65	5	30
25–29	64	12	24	55	10	35
30–34	58	17	25	48	15	37
35–39	58	18	24	46	18	36
40–44	61	15	23	48	19	33
45–49	59	16	25	47	21	32
50–54	56	19	26	45	24	32
55–59	55	21	25	42	27	30
60–64	57	22	21	42	32	26
65–69	64	21	15	45	36	19
70–74	70	21	10	46	40	14
75+	84	12	4	53	40	7
Total	66	16	19	51	23	27

Like Ajrouche et al. (2018) and Simonetto et al. (2021), we utilize data from the SYNERGY study (Pesch et al. 2012) for information on the risk of lung cancer due to smoking. The SYNERGY study combines data from nine case–control studies, with approximately 29% of lung cancer deaths and control persons sourced from Germany. This study provides in Table S10 (Pesch et al. 2012) specific information on the relative risks (RR) for lung cancer risk among current smokers compared to never smokers for the age group of 35–59 years, separately by sex: $RR_S=21.1$ for men (25.5 for men aged 60 and over) and $RR_S=8.1$ for women (7.8 for women aged 60 and over). Corresponding relative risks for former smokers for the age group 35–59 years can also be derived from Table S10 (Pesch et al. 2012): $RR_F=6.1$ for men (9.8 for men aged 60 and over) and $RR_F=3.3$ for women (3.6 for women aged 60 and over).

### Calculation of PAF and attributable deaths

In general, the PAF describes the fraction of risk in a population that is attributable to a specific exposure (Levin 1953) and can be expressed as follows (Miettinen 1974):

2 $$\begin{aligned} PAF = \frac{R-R_0}{R}. \end{aligned}$$

In our analysis, the denominator is the lifetime risk *R* of death from lung cancer in a population subjected to the prevailing residential radon exposure conditions. The numerator represents the potential reduction in risk that could be achieved if radon exposure were eliminated. However, since complete elimination of radon is not feasible, we use an alternative baseline concentration, $x_0$, which is based on the population-weighted outdoor radon concentration (10 Bq/m$^{3}$ in Germany), instead of 0 Bq/m$^{3}$, to calculate the baseline lifetime risk $R_0$ for lung cancer mortality ’without’ radon exposure. In accordance with the WHO’s concept of the global burden of disease, the lifetime risk *R* is adjusted according to the distribution of radon exposure within the population (Prüss-Ustün et al. 2003).

3 $$\begin{aligned} R = \int _0^{\infty } R(x)f(x)dx \approx \sum _{i=0}^W R(x_i)f(x_i). \end{aligned}$$

The relative frequencies $f(x_i)$ of radon exposures $x_i$ are derived from the radon distribution of Petermann et al. (2024). *W* represents the highest possible radon category. Our method for calculating the PAF is similar to that of Menzler et al. (2008) and employs life table methods (Steindorf and Becher 1994) to estimate the lifetime risks *R*(*x*) of dying from lung cancer due to various radon exposures *x*. For more detailed information, please refer to Appendix.

The PAF is computed individually for six demographic subgroups in Germany, divided according to sex (men and women) and the three distinct smoking categories. These calculated PAFs are then multiplied by the annual number of lung cancer deaths in each subgroup to determine the number of deaths attributable to residential radon exposure per year. The sum of these attributable deaths—divided by the total number of annual lung cancer deaths—yields the overall PAF for Germany. In the same way, this calculation method is applied to determine the PAF for each of the 16 federal states of Germany.

When calculating the confidence intervals, we follow the approach of Menzler et al. (2008) and Ajrouche et al. (2018), assuming that all uncertainties in the PAF estimates stem from statistical uncertainties in the parameter estimates of the ERR model. The 95% CI for the PAF is computed by substituting the value of 0.16 in Eq. (1) with both the lower and the upper limits of its 95% CI, namely 0.05 and 0.31.

### Mitigation potential analyses

For radiation protection policy purposes, it is interesting to evaluate how many lung cancer deaths could potentially be prevented by radon mitigation programs. One considered mitigation program aims to reduce home radon levels by remedying dwellings where the radon concentration exceeds a designated threshold. The thresholds considered for action include 100 Bq/m$^{3}$ (recommended by WHO (Zeeb and Shannoun 2009)), 200 Bq/m$^{3}$ (the action level in countries like Ireland, Canada, and the UK (Ruano-Ravina et al. 2017)), 300 Bq/m$^{3}$ (the reference value in the German Radiation Protection Act), and 1000 Bq/m$^{3}$. We explore two scenarios in this context: Scenario 1 assumes that the post-mitigation radon concentration will align randomly with values from the distribution under the threshold. For example, if the threshold value is 300 Bq/m$^{3}$, the new radon concentration assumes a random value between 0 and 300 Bq/m$^{3}$. In Scenario 2, the new radon concentration in a dwelling matches the baseline concentration $x_0=10$ Bq/m$^{3}$. For example, if the threshold value is 300 Bq/m$^{3}$, all radon concentrations of more than 300 Bq/m$^{3}$ are reduced to 10 Bq/m$^{3}$ by remediation. In a third scenario, the effect of reducing radon exposure in all homes by a certain factor (10%, one third, 50%) is investigated. The calculations are performed similar to that described in the previous section, with a key modification in Eq. (5): the lifetime risks are weighted with the respective assumed radon distribution.

### Sensitivity analysis

To investigate the impact of the assumptions made on PAFs due to residential radon exposure and on deaths from lung cancer attributable to radon we repeated the calculations with variations in specific components. The scenarios considered include:

#### Split of total lung cancer deaths to smoking groups

In the main analysis, we employ the splitting method to allocate lung cancer deaths across the three smoking groups, applying it separately for each sex and age group. By aggregating results across age groups, we derive the totals for lung cancer deaths among never smokers, former smokers, and current smokers, categorized by sex. However, these totals could alternatively be obtained by applying the splitting method—separately for each sex—to the overall lung cancer death counts, rather than to age-specific lung cancer death counts. This alternative approach of splitting the total lung cancer deaths, as used by Menzler et al. (2008), is explored in a sensitivity analysis.

#### Smoking specific ERRs due to radon

We repeat our calculations using smoking group-specific ERRs due to radon for the three smoking groups, rather than employing a uniform ERR of 0.16 per 100 Bq/m$^{3}$ corrected long-term radon concentration. For this purpose, we utilize the estimated ERRs provided by Darby et al. (2006): an $ERR= 0.1$ (95% CI < $-$ 0.03 to 0.38) for current smokers, an $ERR = 0.22$ (95% CI 0.02–0.57) for former smokers, and an $ERR = 0.2$ (95% CI 0.02–0.52) for never smokers. These estimates were not found to be statistically significantly different from one another.

#### Approximation of the PAF

We also calculate the PAF using the commonly applied approximation formula based on the average radon exposure $\bar{x}$ (Darby et al. 2005; Gaskin et al. 2018; Gredner et al. 2018). For the linear no-threshold risk model $RR(x)=1+\beta x$, one obtains

4 $$\begin{aligned} PAF \approx \frac{RR(\bar{x})-RR(x_0)}{RR(\bar{x})} = \frac{\beta \cdot (\bar{x} - x_0)}{1 + \beta \cdot \bar{x}}. \end{aligned}$$

#### 2006 radon distribution

We repeat the calculations with the 2006 radon distribution used by Menzler et al. (2008), to estimate the impact of the new radon distribution data.

#### 1996–2000 mortality data

The impact of mortality data is analyzed by employing the same lung cancer mortality and all-cause mortality data for the years 1996–2000 that was used by Menzler et al. (2008).

#### Never smokers vs. ever smokers

Instead of three, we consider only two smoking groups (never smokers and ever smokers) as Menzler et al. (2008).

#### RR for lung cancer due to smoking

The relative risks for lung cancer due to smoking in the age groups 35–54, 55–64, 65–74, and 75+ from the US Surgeon General’s report are utilized (U.S. Department of Health and Human Services 2014). While the estimated relative risks for women are slightly higher than in the SYNERGY study, they are relatively similar for men.

#### Smoking behavior

We assume varying smoking behaviors based on data from the years 1992, 2005, 2009, and 2013 (Menzler et al. 2008; Destatis 2006, 2010, 2014). It is noteworthy that the percentage of smokers in Germany has steadily declined over these years. Specifically, the proportion of current male smokers, which was 40% in 1992, decreased to 31% by 2005 and further to 26% by 2017. Among women, the rates also declined, although starting from a lower initial point and with less substantial decreases (from 27% in 1992 to 22% in 2005, and 19% in 2017). Conversely, the proportion of male never-smokers saw a considerable rise (from 27% in 1992 to 44% in 2005, and 51% in 2017), which was higher compared to the increase among women (from 56% in 1992 to 64% in 2005, and 66% in 2017), yet the proportions for men still have not reached the level observed among women.

#### Future scenarios

We aim to determine the impact of an aging population on the number of lung cancer deaths attributable to radon. For this purpose, we utilize the population forecast provided by the German Federal Statistical Office for the year 2070, assuming moderate settings for birth rates, life expectancy, and migration balance (Destatis 2024). Two hypothetical scenarios are considered: In the first scenario, we assume that age-specific lung cancer rates will remain constant compared to data from 2018–2022. This implies maintaining the same conditions concerning smoking behavior, radon distribution, and levels of medical care. In the second scenario, we explore the potential effects if smoking proportions decrease significantly. Here, we hypothesize an extreme case where, by 2070, the population consists entirely of never smokers. For this group, we apply an ERR of 0.2 per 100 Bq/m$^{3}$ for corrected long-term radon concentration, as estimated by Darby et al. (2006). Since it is estimated that at least 80% of lung cancer deaths are attributable to smoking in Germany (Mons et al. 2018), we reduce the number of age-specific lung cancer deaths by 80% and also reduce the total number of age-specific deaths by 3.6% (representing 80% of the 4.5% proportion of lung cancer deaths among total deaths). These scenarios are designed to evaluate how demographic shifts and changes in smoking prevalence might influence lung cancer mortality associated with residential radon exposure over time.

## Results

### Main results

It is estimated that in Germany approximately 2800 lung cancer deaths per year (95% CI 900–5100) can be attributed to residential radon exposure (Table 3). This represents a PAF of 6.3% (95% CI 2.1–11.4%). Of these 2839 radon-attributable lung cancer deaths, 61% were men and 39% women. 19% occurred among never smokers, 41% among former smokers, and 41% among current smokers. PAFs are highest among never smokers and lowest among current smokers.

**Table 3:** Numbers (N) and percentages (%) of annual lung cancer deaths (LCD) and annual radon-attributable lung cancer deaths (Rn-LCD), as well as PAFs due to residential radon (Rn-PAF) in Germany

Sex	Smoking group	LCD	Rn-LCD		Rn-PAF	
		N (%)	N (%)	95% CI	%	95% CI
Men	Never smokers	1802 (4)	121 (4)	40–218	6.7	2.2–12.1
	Former smokers	13,144 (29)	847 (30)	281–1522	6.4	2.1–11.6
	Current smokers	12,730 (28)	753 (26)	250–1352	5.9	2.0–10.6
	Total	27,676 (62)	1721 (61)	571–3092	6.2	2.1–11.2
Women	Never smokers	6087 (14)	408 (14)	135–734	6.7	2.2–12.1
	Former smokers	4787 (11)	313 (11)	104–563	6.5	2.2–11.8
	Current smokers	6344 (14)	397 (14)	132–714	6.3	2.1–11.3
	Total	17,216 (38)	1118 (39)	371–2011	6.5	2.2–11.7
Total		44,892 (100)	2839 (100)	942–5103	6.3	2.1–11.4

The LCD data correspond to the 5-year average for the years 2018–2022

Table 4 displays the number of radon-attributable lung cancer deaths, PAFs, and mean radon concentrations for the 16 federal states in Germany (Petermann et al. 2024). The lowest PAFs are observed in the city states of Berlin (3.2%), Hamburg (3.3%), and Bremen (3.3%), while the PAF is highest in Thuringia (10.0%), followed by Saxony (9.5%).

**Table 4:** Mean radon concentrations ($\overline{Rn}$), number of annual lung cancer deaths (LCD), number of annual radon-attributable lung cancer deaths (Rn-LCD), and PAFs due to residential radon (Rn-PAF) for the 16 federal states of Germany

Federal State	$\overline{Rn}$	LCD	Rn-LCD		Rn-PAF	
	Bq/m$^{3}$	N	N	95% CI	%	95% CI
Baden-Württemberg	72	4465	317	106–565	7.1	2.4–12.7
Bavaria	85	5234	424	143–746	8.1	2.7–14.3
Berlin	31	2030	64	22–122	3.2	1.1–6.0
Brandenburg	48	1560	78	24–140	5.0	1.5–9.0
Bremen	32	449	15	6–28	3.3	1.3–6.2
Hamburg	30	1034	34	12–66	3.3	1.2–6.4
Hesse	64	3060	193	65–347	6.3	2.1–11.3
Mecklenburg-Western Pomerania	65	1089	71	23–129	6.5	2.1–11.8
Lower Saxony	40	4726	187	61–346	4.0	1.3–7.3
North Rhine-Westphalia	48	11,504	525	171–962	4.6	1.5–8.4
Rhineland-Palatinate	81	2366	195	65–344	8.2	2.7–14.5
Saarland	65	721	47	15–85	6.5	2.1–11.8
Saxony	100	1951	186	64–318	9.5	3.3–16.3
Saxony-Anhalt	82	1545	126	41–222	8.2	2.7–14.4
Schleswig-Holstein	47	2053	98	32–179	4.8	1.6–8.7
Thuringia	103	1132	113	39–194	10.0	3.4–17.1
Germany	63	44,892	2839	942–5103	6.3	2.1–11.4

The LCD data correspond to the 5-year average for the years 2018–2022

### Results of the mitigation potential analyses

The upper part of Table 5 illustrates how many lung cancer deaths could be avoided if all dwellings with a radon concentration above a threshold were remediated to a random radon concentration below the threshold (Scenario 1). For example, remediation of homes with radon levels above 300 Bq/m$^{3}$ could prevent 421 lung cancer deaths, representing 14.8% of radon-induced lung cancer deaths. Additionally, remediating all homes with radon levels above 100 Bq/m$^{3}$ could prevent as many as 966 lung cancer deaths, or 34.0% of radon-induced lung cancer deaths. Further reductions could be achieved under Scenario 2, where radon concentrations are hypothetically reduced to the baseline level of 10 Bq/m$^{3}$ (middle part of Table 5). Of the 2839 radon-attributable lung cancer deaths, 57.8% occur in homes with radon concentrations below 100 Bq/m$^{3}$, 18.0% in the range of 100 to 200 Bq/m$^{3}$, and 24.2% above 200 Bq/m$^{3}$.

**Table 5:** Mitigation potential analysis: the number of avoidable lung cancer deaths (LCD) in Germany by reducing radon concentrations in dwellings with different goals for conducting mitigation measures

Scenario	Reduction of radon concentrations	Threshold [Bq/m$^{3}$]	Avoidable LCD per year	Fraction of all Rn-LCD [%]
1	above the threshold to a random value below the threshold	100	966	34.0
		200	590	20.8
		300	421	14.8
		1000	115	4.1
2	above the threshold to 10 Bq/m^3^	100	1197	42.2
		200	687	24.2
		300	475	16.7
		1000	123	4.3
3	by 10%		272	7.9
	by 1/3		937	27.4
	by 50%		1442	42.1

If alternatively radon concentrations in all dwellings in Germany were reduced by a certain percentage (Scenario 3), a 10% reduction in radon concentrations reduces the number of attributable lung cancer deaths due to radon by 7.9% (lower part of Table 5). Furthermore, this number can be reduced by 27.4% and 42.1% if the radon concentrations in all homes were reduced by one third and one half respectively.

### Results of the sensitivity analyses

Table 6 presents results from the sensitivity analyses. The scenarios ‘Approximation formula’, ‘Never vs. ever smokers (Menzler et al. 2008)’, ‘RR due to US smoking’, and ‘1992 smoking behaviour (Menzler et al. 2008)’ show relatively similar results compared to the main analysis. However, notable differences arise in other scenarios: The analyses for the ‘2006 Rn distribution (Menzler et al. 2008)’ and ‘1996–2000 mortality (Menzler et al. 2008)’ reveal markedly lower numbers of radon-attributable lung cancer deaths, approximately 2,200 and 2,300 respectively, compared to the main analysis. The PAF for ‘2006 Rn distribution (Menzler et al. 2008)’ is significantly lower at 4.9%, while the PAF for ‘1996–2000 mortality (Menzler et al. 2008)’ is similar to the main analysis at 6.2%. Further changes are expected under the assumed scenarios in the future. If lung cancer mortality rates remain constant, radon-attributable lung cancer deaths could increase to around 3300 by the year 2070. If there were only never-smokers by 2070, the number of radon-attributable lung cancer deaths is estimated to decrease by 70% to approximately 900. The total number of radon-attributable deaths in ‘Smoking specific ERRs due to Rn’ is similar to that of the main analysis. However, a larger number of deaths are observed among never smokers and former smokers.

**Fig. 2:**
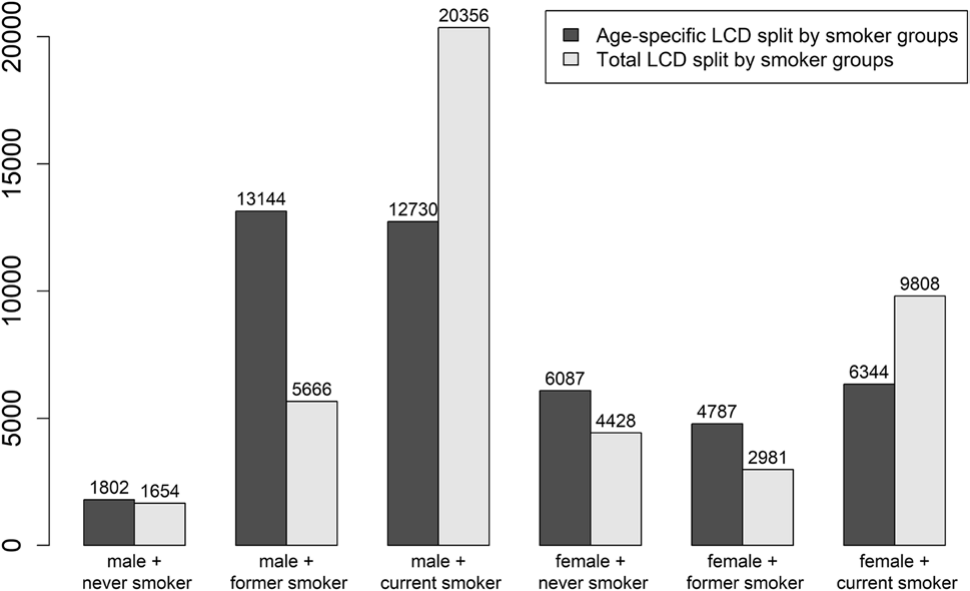
Allocation of lung cancer deaths (LCD) across three smoking groups by sex in Germany using two different methods: splitting the total LCD and splitting age-specific LCD followed by summation

When the total number of lung cancer deaths (‘Total LCD split by smoker groups’), rather than age-specific lung cancer death counts (‘Main analysis’), is distributed among the smoking groups, lung cancer deaths among current smokers increase by 55% for women and 60% for men (Fig. 2). Conversely, lung cancer mortality is estimated to be lower among former smokers (women: by 38%; men: by 57%) and never smokers (women: by 27%; men: by 8%). The same pattern is observed for radon-attributable lung cancer deaths among smoking groups (‘Total LCD split by smoker groups’ in Table 6). Nonetheless, the PAFs, when separated by sex and smoking status, remain consistent across both splitting methods since the calculations only include age- and sex-specific lung cancer rates, and not the actual numbers of lung cancer deaths. Overall, similar totals of radon-attributable lung cancer deaths are reported, regardless of the method used to allocate lung cancer deaths among smoker groups.

**Table 6:** Number of annual radon-attributable lung cancer deaths (Rn-LCD) and PAF due to residential radon (Rn-PAF) as percentages in Germany, as determined by the main analysis and various sensitivity analyses

Analysis	Men						Women						Total	
	Never smokers		Former smokers		Current smokers		Never smokers		Former smokers		Current smokers			
	Rn-LCD	Rn-PAF	Rn-LCD	Rn-PAF	Rn-LCD	Rn-PAF	Rn-LCD	Rn-PAF	Rn-LCD	Rn-PAF	Rn-LCD	Rn-PAF	Rn-LCD	Rn-PAF
Main results	121	6.7	847	6.4	753	5.9	408	6.7	313	6.5	397	6.3	**2839**	**6**.**3**
	40–218	2.2–12.1	281–1522	2.1–11.6	250–1352	2.0–10.6	135–734	2.2–12.1	104–563	2.2–11.8	132–714	2.1–11.4	942–5103	2.1–11.4
Total LCD split by smoker groups	111	6.7	365	6.4	1204	5.9	297	6.7	195	6.5	615	6.3	**2787**	**6**.**2**
	37–200	2.2–12.1	121–656	2.1–11.6	400–2162	2.0–10.6	98–534	2.2–12.1	65–350	2.2–11.8	204–1104	2.1–11.3	925–5006	2.1–11.2
Smoking specific ERRs due to Rn	149	8.2	1129	8.6	486	3.8	500	8.2	417	8.7	257	4.0	**2938**	**6**.**5**
	16–333	0.9–18.5	114–2489	0.9–18.9	$-$ 158–1603	$-$ 1.2–12.6	55–1120	0.9–18.4	42–921	0.9–19.2	$-$ 83–847	$-$ 1.3–13.3	$-$ 14–7313	0.0–16.3
Approximation formula	120	6.7	877	6.7	850	6.7	406	6.7	320	6.7	423	6.7	**2996**	**6**.**7**
	40–216	2.2–12.0	290–1579	2.2–12.0	281–1529	2.2–12.0	134–731	2.2–12.0	106–575	2.2–12.0	140–762	2.2–12.0	991–5392	2.2–12.0
2006 Rn distribution (Menzler et al. 2008)	94	5.2	661	5.0	591	4.6	317	5.2	244	5.1	311	4.9	**2218**	**4**.**9**
	31–172	1.7–9.5	216–1207	1.6–9.2	193–1083	1.5–8.5	104–579	1.7–9.5	80–445	1.7–9.3	102–568	1.6–9.0	726–4054	1.6–9.0
1996–2000 mortality (Menzler et al. 2008)	115	6.7	752	6.4	862	5.7	210	6.7	164	6.6	231	6.5	**2334**	**6**.**2**
	38–206	2.2–12.1	250–1352	2.1–11.4	287–1547	1.9–10.3	70–379	2.2–12.1	54–296	2.2–11.9	77–415	2.2–11.7	776–4195	2.1–11.1
Never vs. ever smokers (Menzler et al. 2008)	97	6.7			1658	6.3	360	6.7			761	6.4	**2876**	**6**.**4**
	32–175	2.2–12.1			550–2978	2.1–11.4	119–647	2.2–12.1			253–1368	2.1–11.5	954–5168	2.1–11.5
RR due to US smoking	152	6.7	724	6.5	857	6.0	236	6.7	296	6.5	560	6.1	**2825**	**6**.**3**
	50–273	2.2–12.1	240–1301	2.2–11.7	285–1539	2.0–10.8	78–426	2.2–12.1	98–532	2.2–11.8	186–1005	2.0–10.9	937–5076	2.1–11.3
1992 smoking behaviour (Menzler et al. 2008)	32	6.7	707	6.6	1034	6.3	368	6.7	247	6.6	505	6.3	**2893**	**6**.**4**
	10–57	2.2–12.1	234–1271	2.2–11.8	343–1858	2.1–11.3	122–661	2.2–12.1	82– 444	2.2–11.8	168–908	2.1–11.4	959–5199	2.1–11.6
2070: LCD rates unchanged	160	6.7	1136	6.4	849	5.9	490	6.7	352	6.5	403	6.3	**3390**	**6**.**3**
	53–287	2.2–12.1	377–2041	2.1–11.6	282–1524	2.0–10.6	162–881	2.2–12.1	117–632	2.2–11.8	134–725	2.1–11.3	1125–6090	2.1–11.4
2070: Only never smokers	547	8.0	0	5.7	0	0.5	311	8.1	0	8.3	0	3.7	**858**	**8**.**0**
	60–1217	0.9–17.7	0	0.6–11.9	0	$-$ 0.2–1.1	34–693	0.9–18.1	0	0.9–18.1	0	$-$ 1.2–11.9	94–1910	0.9–17.9

95% confidence intervals are provided in the respective lower lines. The total results are in bold. The main results are based on the radon distribution of Petermann et al. (2024), mortality data from 2018–2022 (GBE 2023), smoking behaviour data from 2017 (Destatis 2018), and RR for lung cancer due to smoking according to the SYNERGY study (Pesch et al. 2012). In addition, an uniform ERR due to radon is used for the three smoking groups. The splitting method for allocating lung cancer deaths across the three smoking groups is applied separately for each sex and age group

## Discussion

Based on an updated and more precise radon distribution and other updated data the proportion of lung cancer deaths in Germany attributable to radon is calculated to be 6.3% (95% CI 2.1–11.2%) (Table 3). This PAF corresponds to approximately 2800 lung cancer deaths (95% CI 900–5100) annually. Therefore, the number of annual deaths from radon-induced lung cancer in Germany may be roughly equivalent to the total number of annual deaths from malignant melanoma of the skin (ICD10: C43, average = about 2900 for 2016–2020) (ZfKD 2024) or traffic accidents (average = about 2900 for 2018–2022) (Destatis 2024).

The assessment of PAF results at the federal state level in Table 4 reveals significant variations among the German federal states. This is primarily attributed to the differing radon conditions in these states, i.e. the higher the mean radon concentrations the higher the PAFs.

### Comparison with previous findings and other countries

Our approach is similar to Menzler et al. (2008); however, it diverges in two significant ways in addition to some methodological details (see Appendix) and to using new data on radon distribution, lung cancer mortality, smoking behavior, and updated insights on lung cancer risk from smoking. Firstly, we consider three smoking groups instead of two, and secondly, we utilize age-specific data to estimate lung cancer deaths by sex and smoking group. Our current analysis shows a slightly higher PAF and a noteworthy higher number of radon-induced lung cancer deaths in Germany compared to Menzler et al. (2008). The slightly higher PAF (6.3% versus 5.0%) is mainly due to improved knowledge about residential radon distribution in Germany (Petermann et al. 2024), which leads to higher radon values. This is evidenced in the sensitivity analysis using the same radon distribution as in Menzler et al. (2008) (‘2006 Rn distribution’ in Table 6), which resulted in a PAF of 4.9%, almost identical to that in Menzler et al. (2008). Sensitivity analysis ‘1996–2000 mortality (Menzler et al. 2008)’ indicates that the mortality data, on the other hand, have a negligible effect on the PAF values. Both employing the ‘2006 Rn distribution (Menzler et al. 2008)’ and the ‘1996–2000 mortality (Menzler et al. 2008)’ demonstrate that the higher radon levels, coupled with the rise in the annual number of lung cancer deaths over time in Germany, result in an increased number of radon-attributable lung cancer deaths in the current analyses compared to those reported by Menzler et al. (2008).

Other published, rougher estimates for PAF and the number of radon-attributable lung cancer cases in Germany with the approximation formula showed the following pattern: Using incidence data, a similar PAF was calculated with a slightly higher number of radon-attributable cases (e.g. Gredner et al. (2018)). The Global Burden of Disease Study calculated also 2800 (95% CI − 1200 to 8100) radon-attributable lung cancer deaths despite a slightly lower PAF of 5% (95% CI − 2 to 16%) (GBD 2024). However, due to different methodologies and older data used, the results of these studies are difficult to compare with our analysis. Gaskin et al. (2018) reported considerably higher values, e.g. a PAF of 14.9% (95% CI 3.6–29.8%) and 6500 radon-attributable lung cancer deaths (95% CI 1500–13,000) using the BEIR VI risk model.

It is worth noting that PAF estimates derived from risk models based on uranium miner studies, as employed by Gaskin et al. (2018), provide considerably higher values than those based on residential radon studies (Martin-Gisbert et al. 2022).

Estimates of the PAF attributable to radon have also been reported for several other countries, including the United States, Canada, China, South Korea, and Europe. There are three reviews on this subject (Kim et al. 2016; Ajrouche et al. 2017; Martin-Gisbert et al. 2022). In the most recent review, Martin-Gisbert et al. (2022) found that the PAF attributable to radon varied between 3% and 12% in high-quality publications that used residential radon risk models. Since this systematic scoping review, a PAF of 3–8% for Finland (Kurkela et al. 2023) and a PAF of 2.8–6.5% for Slovenia (Birk et al. 2024) have also been published. The differences in the PAF values for different countries are primarily due to differences in the country-specific radon concentrations.

#### Effect of smoking

Among never smokers, a slightly higher proportion of lung cancer deaths is attributable to radon (6.7% for both men and women) compared to former smokers (6.4% for men, 6.5% for women) and current smokers (5.9% for men, 6.3% for women). The differences are relatively small. One might expect a substantially higher proportion of lung cancer deaths attributable to radon among never smokers compared to other smoking categories, since smoking, the predominant risk factor for lung cancer, is not relevant for them. The reason for this discrepancy is primarily due to the application of a uniform ERR of 16% per 100 Bq/m$^{3}$ for all smoking groups, assuming a multiplicative model for the interaction of smoking and radon on lung cancer risk (UNSCEAR 2020), and the use of a uniform radon distribution for all subpopulations.

Since the PAF values in the subpopulations are relatively similar, the distribution of radon-attributable lung cancer deaths by smoking status and sex closely mirrors the distribution of all lung cancer deaths across these groups. If smoking group-specific ERRs from Darby et al. (2006), which are not statistically significantly different, were used, there would be much higher PAFs among never and former smokers compared to current smokers, indicating even more pronounced differences in radon-attributable lung cancer deaths across smoking groups and by sex. Using these smoker-specific parameters, among men, radon-attributable lung cancer deaths are distributed as follows: 8% among never smokers, 28% among current smokers, and, at 64%, predominantly among former smokers. Among women, the majority of radon-attributable lung cancer deaths occur among never smokers (43%), followed by former smokers (36%) and current smokers (22%) (as noted in ‘Smoking specific ERRs due to Rn’ in Table 6). Despite these variations, the total number of attributable lung cancer deaths and the PAF remain relatively similar to those calculated with a uniform ERR. However, the smaller data base across the three smoking groups leads to more uncertainty and wider confidence intervals for ERR, PAF, and the number of attributable deaths. When alternative data for smoking behavior, such as for 1992 (see ‘1992 smoking behaviour (Menzler et al. 2008)’ in Table 6), as well as for the years 2005, 2009, and 2013 (not shown), are used, and when relative risks for lung cancer due to smoking from the US Surgeon General’s report are considered, the results are relatively similar to those in the main analysis (‘RR due to US smoking’ in Table 6)

Examining three rather than two smoking categories reveals very similar outcomes for PAF and the number of lung cancer deaths attributable to radon (as shown in ‘Never vs. ever smokers’ of Table 6). However, this division permits deeper exploration into the interactions between radon and smoking. It is particularly crucial when analyzing current and former smokers separately, to take into account the age dependencies of smoking behaviors (Table 2) and the risks of lung cancer associated with smoking when estimating lung cancer deaths among smoking groups. Neglecting these age dependencies, as in the ‘Total LCD split by smoker groups’ method outlined in Table 6, leads to significantly skewed estimates of both the number of lung cancer deaths and radon-attributable lung cancer deaths in these subpopulations. For example, the radon-attributable lung cancer deaths among current smokers are overestimated by more than half.

#### Mitigation potential analyses

The methodological approach applied here not only enables the calculation of the proportion and number of lung cancer deaths attributable to radon in Germany but also to assess the potential effects of different mitigation measures. Assuming that the radon concentration after mitigation falls at a random value below the threshold, it is estimated that between 115 and 966 lung cancer deaths could be prevented with threshold values ranging from 1000 to 100 Bq/m$^{3}$, respectively. If we consider the rather unrealistic scenario where all radon concentrations after mitigation drop to 10 Bq/m$^{3}$—approximately equivalent to outdoor radon levels—the number of preventable lung cancer deaths could vary from 123 to 1197 for the same threshold values. By reducing radon concentrations in all homes by a third, 27% of radon-attributable lung cancer deaths could be avoided.

Most radon-attributable lung cancer deaths were assigned to individuals living in homes with radon concentrations below 100 Bq/m$^{3}$ since these low radon concentrations are much more common than higher ones and still have a low but non-negligible risk of lung cancer. This low risk is a consequence of the assumed linear risk model without a threshold, which is well supported by residential studies (Darby et al. 2005; UNSCEAR 2020).

A reference level of 300 Bq/m$^{3}$ for the radon concentration in workplaces and living spaces is set out in the German Radiation Protection Act 2021. In homes exceeding this value measures to reduce the radon concentration should be carried out (BfS 2021). If radon concentrations are below this value people are also advised to examine whether the radon concentration can be reduced with reasonable effort or at reasonable expense. Our results give support to these recommendations.

#### Future scenarios

Assuming no changes in radon conditions, smoking behavior, and medical care, an increase in the number of radon-attributable lung cancer deaths is to be expected in the future. Since lung cancer primarily occurs in old age, an aging population leads to more lung cancer deaths overall (estimate for 2070: 19,100 women and 34,400 men). As discussed in the context of sensitivity analysis ‘1996–2000 mortality (Menzler et al. 2008)’, lung cancer mortality data have a negligible effect on the PAF values. Thus, the PAF values remain also unchanged in this scenario. The same PAF values lead to more radon-attributable lung cancer deaths (estimate for 2070: 3400 according to ‘2070: Smoking unchanged’ in Table 6) with more lung cancer deaths occurring.

On the other hand, if everyone were to quit smoking and not start again, the number of lung cancer deaths would drastically decrease. Albeit this would also lead to a decrease in the number of radon-attributable deaths, the estimate would still be 900 deaths according to the scenario ‘2070: Only never smokers’ in Table 6, and the PAF for never-smokers would increase to 8.0%.

#### Strengths and limitations

The main strength of our analyses is that by utilizing new data on radon distribution, lung cancer mortality, and smoking behaviour in Germany, our estimates for the PAF and the number of radon-attributable lung cancer deaths are up-to-date.

Another strength is that our calculation method considers the entire distribution of radon exposure instead of only the mean radon exposure as in the approximation formula (4) often used in the literature (Darby et al. 2005; Gaskin et al. 2018; Gredner et al. 2018). Generally, Eq. (2) combined with Eq. (3) can be transformed into Eq. (4) if $ERR_k(x)$ were age-independent and if the probability of reaching a certain age group *k* is independent of radon exposure *x*. However, due to existing dependencies, this approximation results in a slight overestimation of the PAF. Indeed, for Germany, the PAF increases from 6.3 to 6.7% with the average radon concentration $\bar{x}= 63$ Bq/m$^{3}$ and the baseline radon concentration $x_0= 10$ Bq/m$^{3}$, leading to an increase in the number of radon-attributable lung cancer deaths from approximately 2800 to 3000.

Additionally, our method uses age-specific data on lung cancer mortality, smoking behavior, and lung cancer risk due to smoking. This leads to a more accurate allocation of attributable lung cancer deaths across different smoking groups.

However, both the calculated number of radon-attributable lung cancer deaths and the calculated corresponding fraction have relatively large confidence intervals. In addition, these estimates are associated with uncertainties related to several factors: the assumed risk model for the relationship between lung cancer risk and residential radon exposure (Darby et al. 2006), the presumed multiplicative interaction between radon and smoking on lung cancer risk, the estimated prevalence of smoking, the assumed lung cancer risks due to smoking, and the assumption that other lung cancer risk factors such as particulate matter, asbestos or secondhand smoke do not modify the effect of radon on lung cancer (ICRP 2007).

## Conclusion

The results, based on updated data and refined methodology, confirm that radon in homes is a significant risk factor for lung cancer, with 2800 radon-attributable lung cancer deaths per year and a radon-attributable fraction of 6.3%. These findings underscore the importance of implementing protective measures against radon across Germany for all population groups. A substantial number of radon-attributable lung cancer deaths could be avoided by reducing radon exposure in all homes, including those with radon concentrations below the reference value, as far as practicably achievable with reasonable effort and cost.

## Data Availability

No datasets were generated or analysed during the current study.

## Appendix: Calculation of PAF: details

The PAF is determined similar to Menzler et al. (2006) using Eqs. (2) and (3). The lifetime risk *R*(*x*) of death from lung cancer due to radon exposure *x* can be expressed as a function of the age-specific lung cancer mortality rates $m_k$ and the age-specific mortality rates for all causes of death $m_k^{*}$ using the ERR in Eq. (1):

$$\begin{aligned} R(x)&\approx \sum _{k=1}^K q_k(x) \prod _{j=1}^{k-1} (1-q_j^{*}(x)) \approx \sum _{k=1}^K \frac{m_k(x)}{m_k^{*}(x)} q_k^{*}(x) \prod _{j=1}^{k-1} (1-q_j^{*}(x)).\\ m_k(x)&= m_k \cdot \frac{1 + ERR_k(x)}{1 + ERR_k(\bar{x})} \\ m_k^{*}(x)&= m_k^{*} - m_k + m_k(x) \\ q_k^{*}(x)&= 1 - \exp (-l_k \cdot m_k^{*}(x)) \end{aligned}$$

Here, *q* (or *m*) is the probability (or rate) of dying from lung cancer in age group *k*, given it was reached. The superscript $*$ denotes the corresponding probability of death or mortality rate for all causes of death. The lung cancer mortality rate for mean radon exposure $m_k(\bar{x})$ corresponds to the general lung cancer mortality rate in the population $m_k$. Here, we deviate from the calculation method by Menzler et al. (2006), which uses the general lung cancer mortality rate $m_k$ as the baseline risk for lung cancer $m_k(0)$. This different approach has a negligible effect on PAF, but a substantial effect on the calculation of the lifetime risk *R*. The formula for $q_k^{*}(x)$ is a generalization of Menzler et al. (2006) for arbitrary lengths of age classes $l_k$.

For calculating the lifetime risk $R_0$, a fictive radon exposure distribution $f^{*}$ is assumed in which all corrected long-term radon concentrations above the radon class *I* containing the baseline radon concentration $x_0$ was reduced to this class in all dwellings:

5 $$\begin{aligned} R_0 \approx \sum _{i=0}^{I} R(x_i)f^{*}(x_i). \end{aligned}$$

Again, we differ from Menzler et al. (2008)’s calculation approach, which uses $R_0=R(x_0)$. However, our somewhat more intuitive approach leads to practically the same result.
